# Supplementary material for: Patterns of Intron Gain and Loss in Fungi
Source: PLoS Biol. 2004 Nov 30;2(12):e422. doi: 10.1371/journal.pbio.0020422 (PMC532390; doi:10.1371/journal.pbio.0020422)
Supplement: Table S1 — Also available at http://genes.mit.edu/NielsenEtAl/. (4.3 MB ZIP). [file pbio.0020422.st001.zip › NielsenEtAl/html/215.html]

AN1191.1.NCU09813.1.MG05737.1.FG01322.1


```
 CLUSTAL W (1.82) Multiple Sequence Alignments - Introns Inserted


Sequence 1: AN1191.1	94 aa
Sequence 2: FG01322.1	98 aa
Sequence 3: NCU09813.1	99 aa
Sequence 4: MG05737.1	109 aa
Alignment Length: 111 aa
Number Identitical Residues: 63 aa
Alignment Score (without introns) 2559


MG05737.1 	MSDRENGGSPHGDYPGAGGGAPAGGGDPAAPEHLNIKVTDNNNEVFFKIKRSTKLEKLMN
NCU09813.1	MADNAENGSGN-----AGSDAAAP-----AVEHLNIKVTDNNNEVFFKIKRSTKLEKLMT
FG01322.1 	MSDENANGTPG-------EQAPAN------SEHLNIKVTDNNNEVFFKIKRTTKLEKLMG
AN1191.1  	MSDPSAP-TP---------EAPAP------VEHLNIKVTDNNNEVFFKIKRTTTLKKLMD
          	*:*     :           *.*        ********************:*.*:*** 

MG05737.1 	AFCDRQGKSLSQVRFLFDGQRVQPTDTPDT0LEMADGDTLEVHQEQVGGA--
NCU09813.1	AFCERQGKTLASVRFLFEGQRVQPTDTPDT0LEMQDGDCLEVHQEQVGGW--
FG01322.1 	AFCERQGKATSSVRFLFDGTRVQPTDTPDA0LEMQDGDTLEVHQEQVGGSSL
AN1191.1  	AFCDRQGKQPSTVRFLFDGTRVRPEDTPDT0LDMADGDTLEVHQEQIGGG-L
          	***:****  : *****:* **:* ****: *:* *** *******:**
```
